# A Genome-Wide Analysis of the Penumbral Volume in Inbred Mice following Middle Cerebral Artery Occlusion

**DOI:** 10.1038/s41598-019-41592-5

**Published:** 2019-03-25

**Authors:** Robert F. Rudy, Nareerat Charoenvimolphan, Baogang Qian, Annerose Berndt, Robert M. Friedlander, Scott T. Weiss, Rose Du

**Affiliations:** 1 Department of Neurosurgery, Brigham and Women’s Hospital, Boston, Massachusetts, USA; 2 Harvard Medical School, Boston, Massachusetts, USA; 3 Division of Pulmonary, Allergy, and Critical Care Medicine, University of Pittsburgh School of Medicine, Pittsburgh, Pennsylvania USA; 4 Department of Neurosurgery, University of Pittsburgh School of Medicine, Pittsburgh, Pennsylvania USA; 5 Channing Division of Network Medicine, Brigham and Women’s Hospital, Boston, Massachusetts, USA

## Abstract

Following ischemic stroke, the penumbra, at-risk neural tissue surrounding the core infarct, survives for a variable period of time before progressing to infarction. We investigated genetic determinants of the size of penumbra in mice subjected to middle cerebral artery occlusion (MCAO) using a genome-wide approach. 449 male mice from 33 inbred strains underwent MCAO for 6 hours (215 mice) or 24 hours (234 mice). A genome-wide association study using genetic data from the Mouse HapMap project was performed to examine the effects of genetic variants on the penumbra ratio, defined as the ratio of the infarct volume after 6 hours to the infarct volume after 24 hours of MCAO. Efficient mixed model analysis was used to account for strain interrelatedness. Penumbra ratio differed significantly by strain (F = 2.7, P < 0.001) and was associated with 18 significant SNPs, including 6 protein coding genes. We have identified 6 candidate genes for penumbra ratio: *Clint1*, *Nbea*, *Smtnl2*, *Rin3*, *Dclk1*, and *Slc24a4*.

## Introduction

Ischemic cerebrovascular disease is a leading cause of death globally, accounting for approximately 6.5 million deaths worldwide^1^, and is projected to increase in incidence over the coming decades^2^. Upon large cerebral artery occlusion, an area of core neural tissue infarcts within minutes while tissue surrounding the core, termed the penumbra, persists in an at-risk state. Within the penumbra, diverse and competing influences mediate whether this tissue will eventually infarct^3^. Recent large randomized clinical trials have demonstrated the success of intra-arterial clot removal within a specified time interval in rescuing not-yet-infarcted penumbra^4–6^. While this time window has increased in recent trials^7,8^, there are still restrictive requirements for thrombectomy, with only 7% of patients with ischemic stroke that were eligible for thrombectomy in 2016^9^. Recently, the appreciation of fast and slow progressors after large vessel occlusion has prompted discussion of future trials using imaging data to determine who may benefit from treatment^10^. This heterogeneity in response to ischemia suggests the time windows used currently may not predict outcome accurately in all comers. Thus, understanding factors that affect the penumbra is important in determining in whom and at what time intervention would be beneficial after large artery occlusion.

Discrepancy in infarct volume between strains following middle cerebral artery occlusion (MCAO) is associated with both vascular and neural variation. In particular, differences in infarct size across mouse strains is associated with native arterial collateralization^11–14^, incomplete circle of Willis^15,16^, and susceptibility of neurons to ischemia^17–21^. However, the question of infarct volume is different from that of the proportion of penumbra to core infarct and is particularly pertinent in the era of endovascular stroke therapy. In this study, 449 male mice spanning 33 strains underwent middle cerebral artery occlusion (MCAO). Each strain contains a unique set of single nucleotide polymorphisms and by comparing the penumbra volume in each strain using a genome-wide analysis, we identified genetic polymorphisms associated with penumbral size.

## Methods

### Animal Use Statement

All animal care, housing, and experiments in this study were approved by and conducted in accordance with the PHS Policy on Humane Care and Use of Laboratory Animals from the Institutional Animal Care and Use Committee of the Harvard Medical Area Standing Committee on Animals. Experiments are reported in accordance with the ARRIVE guidelines.

### Middle Cerebral Artery Occlusion Model

33 inbred strains from the Jackson Laboratory were included in this study. The mouse strains were chosen for genetic diversity^22^ and include mice that belong to 6 major groups: 1) Bagg albino (A/J, AKR/J, BALB/cJ, C3H/HeJ, CBA/J, CE/J, LG/J, MRL/MpJ, PL/J), 2) Swiss (BuB/BnJ, FVB/NJ, MA/MyJ, NOD/ShiLtJ, RIIIS/J, SJL/J, SWR/J), 3) Japanese and New Zealand (KK/HlJ, NON/ShiLtJ, NZO/HlLtJ, NZW/LacJ), 4) C57-related (C57BL/6J, C57BL/10J, C57BLKS/J, C57BR/cdJ, C57L/J), 5) Castle (129S1/SvImJ, BTBR T^+^ tf/J, LP/J), and 6) DBA (DBA/1J, DBA/2J, I/LnJ, P/J, SM/J) strains^23^. 449 mice underwent MCAO as previously described^17^. The number of mice excluded for each strain due to unsuccessful surgery or death for the 6-hour MCAO is shown in Supplementary Table 1. Animals excluded for the 24-hour model were previously reported^17^. Eight- to 10-week-old mice were used for the MCAO model. Briefly, mice were anesthetized with 1–3% isoflurane and a 7.0 monofilament threaded into the internal carotid artery to access the middle cerebral artery. Successful occlusion of the middle cerebral artery was defined as ≥80% decrease in cerebral blood flow (CBF) as measured by laser Doppler (Perimed, Jarfalla, Sweden) when comparing the CBF before and after MCAO. Animals were sacrificed by cervical dislocation under 1–3% isoflurane anesthesia. 215 were sacrificed 6 hours after MCAO, and the remaining 234 were sacrificed 24 hours after MCAO. The number of mice per strain at each time point along with age, weight, and mean arterial pressure are documented in Supplementary Table 2.

### Evaluation of Infarct Volume

At the time of sacrifice, the brain was removed from the skull and sectioned into 1 mm coronal slices using a brain matrix. Slices were incubated in 2% triphenyltetrazolium (TTC, Sigma, St. Louis, MO) for 15 minutes at room temperature. Infarct volume was assessed by an investigator blinded to the genetics data at the time of measurement. Infarct volume was obtained by calculating the sum of infarct areas across all slices. Normalized infarct volumes were calculated using the indirect Swanson method: (contralateral volume – ipsilateral noninfarcted volume)/contralateral volume^24^. The penumbra ratio was defined as the ratio of normalized infarct volume at 6 hours to that of the mean normalized infarct volume at 24 hours across all mice in that particular strain. Data for the 24-hour time point were previously reported^17^. Strains with a penumbra ratio closer to one were considered to have a smaller penumbral volume as compared to strains with a lower ratio.

### Association Between Circle of Willis Completeness and Penumbra Ratio

We have previously published the results of an analysis of circle of Willis variation between mouse strains^25^. Using these data, we calculated the average number of P1 segments per strain. We used the average P1 per strain value as a surrogate for completeness of the circle of Willis. A Spearman correlation was used to analyze the correlation between circle of Willis completeness with penumbra ratio.

### Genome-Wide Association Analysis

Following volumetric assessment of MCA territory infarcts, two genome wide association analyses were performed using normalized infarct volume at 6 hours and penumbra ratio as the phenotypes. 132,285 SNPs from the NCBIM37 mouse genotype build were obtained from the UCLA and Broad Institute Mouse HapMap project (http://mouse.cs.ucla.edu/mousehapmap/). SNP data were converted to a numerical format with the R package *GAPIT*^26^. SNPs that were absent in greater than 10% of strains were excluded. Minor allele frequency (MAF) was calculated using Plink v1.9^27^ (http://pngu.mgh.harvard.edu/purcell/plink/), with the major allele defined as the most frequent allele at each location amongst the strains included in this study. SNPs with a MAF of ≤0.05 were excluded. A total of 104,890 SNPs remained after filtering. The efficient mixed model association method was utilized, which accounts for inbred strain interrelatedness and population structure in mice, to generate a genome-wide association map (*emma* package in R^28^). Covariates were excluded on the basis of univariate regression P values > 0.1. These data were then visualized with Manhattan and QQ plots using the *qqman* package^29^. P values were adjusted for multiple testing using the Benjamini and Hochberg false discovery rate (FDR) algorithm^30^. FDR less than 0.05 were considered statistically significant. The genetic location of each SNP was obtained using the UCSC NCBIM37 (mm9) database^31,32^. Protein coding genes within 500 kbp of significant SNPs were identified using BiomaRt^33^ since the majority of enhancers fall within this window^34^. A network analysis was conducted on genes associated with significant SNPs using StringDB v10.5^35^ (medium confidence threshold, all interaction sources, https://string-db.org/). Pathway overrepresentation analysis was performed using the package *WebGestaltR*^36^, with modifications, to search for pathways in KEGG^37^ (10/1/2016 release), Reactome^38^ (version 2016), and Wikipathway^39^ (5/10/18 release), for targets of miRNA and transcription factors from MSigDB^40^ (v6.0, 2017), and for protein-protein interaction networks from BioGRID^41^.

### Power calculations

We performed simulations to analyze the statistical power using the package *emmaPowerSim*^42^ with modifications to account for replicates. The simulation assumes an average minor allele frequency of the causal SNP to be 0.3 and a background genetic effect size of 0.1. The simulation was performed in the 33 strains of mice used in this study for 100 causal SNPs, using 2, 4, 6, or 8 replicates per strain, and varying SNP effect size from the causal SNPs. The power analysis demonstrated that 6 replicates per strain are needed to achieve a power of 80% for a SNP effect of 0.4 (Supplementary Fig. 1). Furthermore, additional replicates only resulted in incremental increases in power. We therefore included a median of 6 technical replicates per strain.

### Statistical Analysis

Statistical analysis was performed using R v3.4^43^. Wilcoxon rank sum (Mann-Whitney *U*) test was used to assess differences in the infarct volume at 6 and 24 hours. ANOVA of univariate linear regression models were used to assess for inter-strain variability for each outcome. Univariate linear regression was used to screen covariates for inclusion in the mixed model. The *ggrepel*^44^, *tidyr*^45^, *plyr*^46^, *dyplr*^47^, *broom*^48^, *data.table*^49^, *reshape2*^50^, *RColorBrewer*^51^, and *ggplot2*^52^ packages were used for data preparation and visualization. Raw images of TTC stained brains were not modified with the exception of resizing of the whole image and cropping for purposes of organization into figures. Figures were assembled in Adobe Illustrator (Adobe Systems, San Jose, CA).

## Results

### Descriptive Analysis of Mouse Characteristics

**Figure 1:**
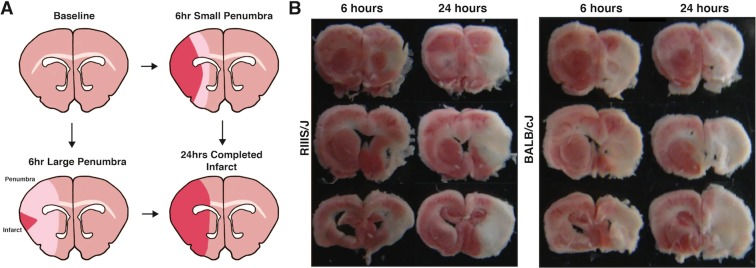
Representative brain slices. (**A**) Graphical representations and (**B**) photographs of penumbral infarction variation in coronal mouse sections at 6 and 24 hours post MCAO stained with 2% triphenyltetrazolium (TTC). RIIIS/J mice have small and BALB/cJ mice have large penumbra ratios.

A total of 449 male mice from 33 strains that underwent MCAO were included in this study, 215 of which were sacrificed at 6 hours and 234 of which at 24 hours (Table 1, stratified by strain in Supplementary Table 2). Mouse strains had significantly varied infarct volume at 6 hours (F = 9.74, P < 0.001), infarct volume at 24 hours (F = 8.54, P < 0.001), and penumbra ratio (F = 2.7, P < 0.001). Mean arterial pressure (MAP), weight, and age of mice sacrificed at 6 and 24 hours also varied between strains (Supplementary Table 3). However, these were not significantly associated with normalized infarct volume using univariate linear regression at their respective time points and were not significantly associated with penumbra ratio at 6 hours (Supplementary Table 3). Representative images of TTC staining in strains with relatively small (RIIIS/J) and large (BALB/cJ) penumbra ratios are presented in Fig. 1. Supplementary Figs 2A and 3A depict the distribution of normalized infarct volumes at 6 hours and penumbra ratio by strain, respectively.

**Table 1:** Characteristics of mice subjected to MCAO.

Characteristics	6-Hour MCAO Median (IQR) (N = 215)	24-Hour MCAO Median (IQR) (N = 234)	P*
Age (weeks)	8.3 (8.1–8.4)	8.2 (8.0–8.30)	0.28
Weight (grams)	24.7 (22.4–27.7)	23.7 (22.7–27.5)	0.02
Mean Arterial Blood Pressure (mmHg)	84.7 (77.5–90.3)	82.8 (77.7–87.7)	0.51
Normalized Infarct Volume	0.55 (0.44–0.61)	0.63 (0.55–0.69)	<0.001

MCAO = middle cerebral artery occlusion, IQR = interquartile range.

*Derived from Wilcoxon rank sum test between non-averaged characteristics at 6 and 24 hours.

### Correlation Between Circle of Willis Completeness and Penumbra Ratio

To assess the impact of variation in circle of Willis completeness, we investigated the association between the average number of P1 segments with penumbra ratio. P1 presence was previously reported by our group in 144 mice across the same 33 strains^25^. Using these data, the average number of P1s per strain did not correlate with penumbra ratio (ρ = 0.10, P = 0.56) (Supplementary Fig. 3). Lack of correlation between P1 presence, and hence circle of Willis completeness, enables analysis of the penumbra ratio with less concern for significant confounding from anatomic variation in the circle of Willis.

### Genes Associated with Infarct Size at 6 hours

24 genome-wide significant SNPs were identified to be associated with the 6-hour normalized infarct volume (Supplementary Fig. 2B, Supplementary Table 5). QQ plot for the model demonstrated no notable deviation from expectation at high p values (Supplementary Fig. 2C). One of the significant SNPs, rs3677406, was previously reported by our group to be significant for the 24-hour normalized infarct volume^17^. Significant SNPs were located within four unique protein-coding genes – *Tshz3*, *Zfp536*, *Ctif*, And *Clint1* – which did not have any associations in StringDB^35^ or pathway/target overrepresentation using *WebGestaltR*^36^.

### Genes Associated with Penumbra Ratio

**Figure 2:**
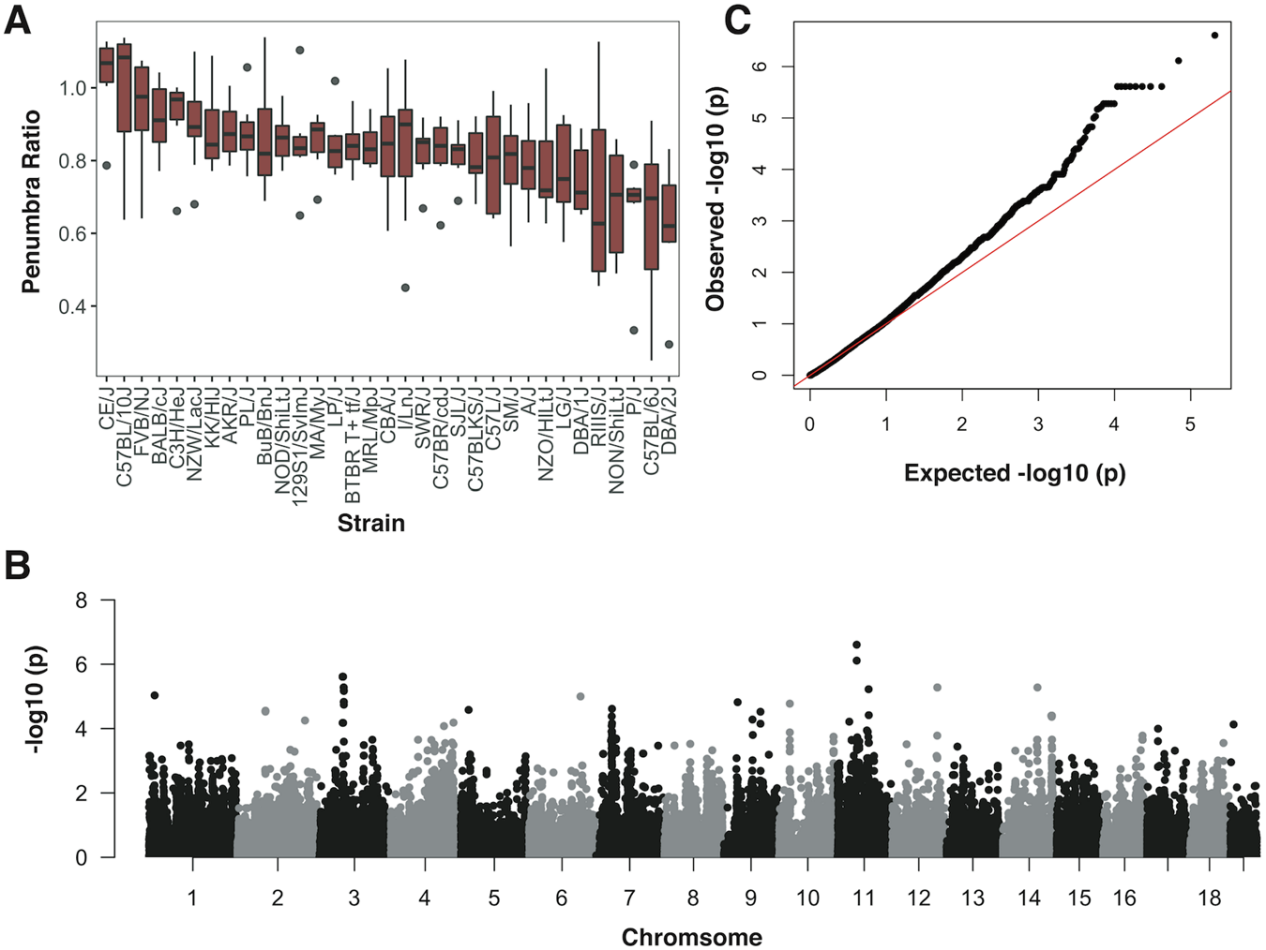
Summary of penumbra ratio by strain and results from genome-wide association analysis. (**A**) Box plot illustrating distribution of normalized infarct ratios sorted by strain. (**B**) Manhattan plot for normalized infarct ratio. (**C**) QQ plot for the penumbra ratio.

18 SNPs spanning four chromosomes and encompassing 6 protein coding genes were significantly associated with penumbra ratio following FDR correction (Fig. 2B, Table 2). QQ plot of the model demonstrated no notable deviation from expectation at high p values (Fig. 2C). The six protein coding genes – *Clint1*, *Nbea*, *Smtnl2*, *Rin3*, *Dclk1*, And *Slc24a4* – did not have any interactions in StringDB^35^ or pathway/target overrepresentation using *WebGestaltR*^36^. However, 4 of the 6 significant genes (*Dclk1, Slc24a4, Nbea, Smtnl2*) are targets of the Myod1 transcription factor and 3 of the 6 significant genes (*Dclk1, Clint1, and Slc24a4*) are associated with miR-145. Two SNPs – rs31452396 and rs13472659 – were significantly associated with both infarct volume at 6 hours and penumbra ratio, one of which resides within *Clint1* (rs13472659).

**Table 2:** 18 significant SNPs (FDR <0.05) associated with penumbra ratio are located within 6 protein coding genes.

SNP	Chr	BP	Major/Minor Allele	MAF	Percent Missing^*^	Protein Coding Gene Containing SNP	SNP location^†^	Nearby Protein Coding Genes^‡^ (distance in BP)	P	FDR
rs30574784	3	55157811	A/G	0.11	0	*Dclk1*	Intron	*Ccna1* (298834), *Spg20* (216567), *Mab21l1* (428621), *Nbea* (271316), *Sohlh2* (143932)	2.44 × 10^−6^	2.56 × 10^−2^
rs30574795	3	55162055	T/C	0.11	0	*Dclk1*	Intron	*Ccna1* (303078), *Spg20* (220811), *Mab21l1* (424377), *Nbea* (267072), *Sohlh2* (148176)	2.44 × 10^−6^	2.56 × 10^−2^
rs30582194	3	55186555	C/T	0.11	0	*Dclk1*	Intron	*Ccna1* (327578), *Spg20* (245311), *Mab21l1* (399877), *Nbea* (242572), *Sohlh2* (172676)	2.44 × 10^−6^	2.56 × 10^−2^
rs30580433	3	55285182	T/G	0.11	0	*Dclk1*	Intron	*Ccna1* (426205), *Spg20* (343938), *Mab21l1* (301250), *Nbea* (143945), *Sohlh2* (271303)	2.44 × 10^−6^	2.56 × 10^−2^
rs30581357	3	55285513	T/C	0.11	0	*Dclk1*	Intron	*Ccna1* (426536), *Spg20* (344269), *Mab21l1* (300919), *Nbea* (143614), *Sohlh2* (271634)	2.44 × 10^−6^	2.56 × 10^−2^
rs30311573	3	55299952	G/A	0.11	0	*Dclk1*	Intron	*Ccna1* (440975), *Spg20* (358708), *Mab21l1* (286480), *Nbea* (129175), *Sohlh2* (286073)	2.44 × 10^−6^	2.56 × 10^−2^
rs30622394	3	55669318	C/T	0.11	0	*Nbea*	Unknown	*Mab21l1* (80111), *Dclk1* (326332)	2.44 × 10^−6^	2.56 × 10^−2^
rs30662015	3	56120522	A/G	0.11	0		Unknown	*Nbea* (132899)	2.44 × 10^−6^	2.56 × 10^−2^
rs31530387	3	57429581	G/A	0.12	0		Unknown	*Tm4sf1* (323740), *Tm4sf4* (183982), *Wwtr1* (49749), *Commd2* (17245), *Rnf13* (110412), *Pfn2* (216236)	5.28 × 10^−6^	3.69 × 10^−2^
rs30762877	3	57470472	C/T	0.12	0		Downstream Gene Variant	*Tm4sf1* (364631), *Tm4sf4* (241236), *Wwtr1* (90640), *Commd2* (14835), *Rnf13* (69521), *Pfn2* (175345)	5.28 × 10^−6^	3.69 × 10^−2^
rs30309818	3	57807137	C/T	0.09	6.1		Unknown	*Wwtr1* (427305), *Commd2* (351500), *Rnf13* (167790), *Pfn2* (155651), *Tsc22d2* (412300)	6.47 × 10^−6^	3.95 × 10^−2^
rs31452396	3	57807198	C/T	0.12	3.0		Unknown	*Wwtr1* (427366), *Commd2* (351561), *Rnf13* (167851), *Pfn2* (155712), *Tsc22d2* (412239)	6.78 × 10^−6^	3.95 × 10^−2^
rs29395502	11	45685166	A/G	0.20	0	*Clint1*	Intron	*Lsm11* (56605), *Thg1l* (75179), *Sox30* (108646), *Adam19* (182838), *Nipal4* (276491), *Cyfip2* (322191), *Itk* (453486)	7.70 × 10^−7^	2.56 × 10^−2^
rs13472659	11	45715732	C/T	0.18	3.0	*Clint1*	Synonymous Coding	*Lsm11* (26039), *Thg1l* (44613), *Sox30* (78080), *Adam19* (152272), *Nipal4* (245925), *Cyfip2* (291625), *Itk* (422920), *Fam71b* (494329)	2.47 × 10^−7^	2.56 × 10^−2^
rs29467394	11	72210606	G/T	0.12	6.1	*Smtnl2*	Intron	*Aipl1* (359595), *Fam64a* (349734), *Pitpnm3* (261326), *Txndc17* (187089), *Med31* (181512), *Slc13a5* (129882), *Xaf1* (83371), *Fbxo39* (77688), *Tekt1* (34662), *Ggt6* (38422), *Mybbp1a* (44251), *Spns2* (54534), *Spns3* (97815), *Ube2g1* (210179), *Ankfy1* (292902), *Cyb5d2* (380128), *Zzef1* (399122)	5.98 × 10^−6^	3.92 × 10^−2^
rs29195737	12	103470716	T/A	0.12	0	*Slc24a4*	Intron	*Fbln5* (413451), *Trip11* (319239), *Atxn3* (274260), *Cpsf2* (226513), *Rin3* (50542), *Lgmn* (161578), *Golga5* (237404), *Chga* (322463), *Itpk1* (336077)	5.28 × 10^−6^	3.69 × 10^−2^
rs29199040	12	103599807	G/A	0.12	0	*Rin3*	Intron	*Trip11* (448330), *Atxn3* (403351), *Cpsf2* (355604), *Slc24a4* (94506), *Lgmn* (32487), *Golga5* (108313), *Chga* (193372), *Itpk1* (206986), *Moap1* (378233), *Ubr7* (396370). *Btbd7* (423059)	5.28 × 10^−6^	3.69 × 10^−2^
rs31307500	14	85181306	C/T	0.12	0		Unknown	*Pcdh17* (244439)	5.28 × 10^−6^	3.69 × 10^−2^

Chr = chromosome, BP = base pair, FDR = false discovery rate, MAF = minor allele frequency.

*Percent of strains missing information at that SNP.

^†^SNP function from UCSC mm9 genome browser.

^‡^Nearby genes within 500 kbp excluding cDNA and predicted genes.

## Discussion

In this study, we investigated the size of the ischemic penumbra at 6 hours by normalizing 6-hour infarct volumes to strain averaged infarct volumes at 24 hours, at which point the infarct has likely completed. Prior studies have identified SNPs associated with infarct volume at 24 hours and have linked those SNPs to genes potentially involved in stroke susceptibility^17,19–21^. Keum *et al*. used an inbred mouse genome wide approach to identify quantitative trait loci on chromosome 7, *Civq1*, as an important determinant of infarct volume^19^. The group subsequently identified *Itgal1*^20^ and *Il-21r*^21^ as candidate genes associated with this region and has also reported additional loci contributing to variation in infarct volume amongst mice^53^. Our own group has previously reported an integrated analysis using inbred mice and human data to identify human *ANGPT1* and *ZBTB7C* as candidate genes associated with middle cerebral artery infarct volume^17^.

However, the question of penumbra size has not previously been addressed. By dividing the normalized 6-hour infarct volume, which has previously been used to study penumbral gene expression^54^, by that of the average normalized volume at 24 hours, we generated a ratio of how much tissue had infarcted between 6 and 24 hours relative to the final infarct volume at 24 hours, which is representative of the penumbra. We utilized a proximal MCAO model that has the advantage of avoiding challenges with variations in distal MCA anatomy, but the final infarct volume may be affected by completeness of the circle of Willis^15,16^. To address this, we analyzed the association between P1 presence and penumbra ratio. The absence of a correlation suggests the penumbra volume calculated in this study is not dependent on circle of Willis completeness. The significant variation in the penumbra ratio across strains suggests that there is a genetic predisposition to penumbral size. This is, perhaps, not surprising, given the variation in infarct volume across strains^11–18^.

Of the 24 significant SNPs associated with infarct volume at 6 hours, one (rs3677406 on the *Ctif* gene) had been previously identified by our group to be associated with infarct volume at 24 hours^17^ whereas the remainder were unique to the 6-hour time point. It is plausible that the determinants of the size of the initial infarct core at 6 hours is not associated with that of the final infarct volume at 24 hours which is a function of both the core and the penumbra, therefore we would expect some of the associated SNPs to be unique to each time point. Of note, we also previously found the *Ctif* gene to be significantly associated with infarct volume in human stroke patients^17^. CTIF has been hypothesized to function as a translation initiation factor in the pioneer phase of translation, which appears to be the predominant mechanism by which mRNA is translated in the setting of hypoxia^55^. It follows that CTIF abnormalities could contribute to ischemic injury via decreased pioneer translation capacity.

To address the question of penumbral size, we performed a genome-wide association analysis with penumbra ratio as the outcome. The 18 SNPs significantly associated with penumbra volume were located within with 6 protein coding genes (*Clint1, Dclk1, Slc24a4, Rin3, Smtnl2*, And *Nbea*). The majority of known SNPs were found to be in introns, which suggests they may be involved in regulation of expression rather than in structural differences of the resulting protein. Two of the SNPs significant for penumbra volume were also significant at the 6-hour time point, rs31452396 and rs13472659, the latter of which is in *Clint1*. While rs13472659 is a synonymous coding SNP, it was found to be significant in two separate analyses is suggestive of its relevance to cerebral ischemia. CLINT1 (also called EPN4) is enriched in the brain and functions in the endocytosis of clathrin coated pits^56^. *Clint1* mutant zebrafish have a phenotype mimicking psoriasis, suggesting it may function in mediating inflammation^57^. Genetic polymorphisms that result in dysregulated inflammatory responses to ischemia could potentiate the inflammatory penumbra, a concept proposed to account for secondary brain injury outside the initial ischemic territory^58^. Furthermore, zebrafish with *Clint1* mutations have increased matrix metalloproteinase 9 (MMP-9) expression^59^. MMP-9, a proteolytic enzyme that normally functions in extracellular matrix remodeling that has been shown to function in blood brain barrier function, increases the degree of ischemic cerebral injury following stroke^60–63^. Increased permeability of the blood brain barrier enables immune cell extravasation and may exacerbate secondary brain injury. Another protein identified in this study, RIN3, a RAB5 guanine exchange factor, functions in early endocytosis^64^ and acts as a negative regulator in mast cells^65^. Mast cells in turn mediate early peri-infarct inflammation following MCAO in rats via blood brain barrier dysfunction and subsequent inflammatory cell extravasation^66^, potentially contributing to the inflammatory penumbra.

*Clint1* expression has also been reported to increase in male, but not female, C57BL/6 mice following MCAO^67^, suggesting male and female mice might respond differently to ischemic injury. All mice included in this study were male, so our results are consistent with the study from Lusardi *et al*.^67^. Interestingly, expression of another gene associated with SNPs significantly associated with penumbra ratio, *Dclk1*, which encodes a kinase associated with microtubule polymerization^68^, has been shown to increase in ischemic female rats following the inhibition of let-7f, a microRNA that suppresses insulin-like growth factor 1 translation^69^. This effect was not observed in male or female rats following oophorectomy, suggesting this pathway is dependent on estrogen and hinting that DCLK1 activity in the setting of cerebral ischemia may have a protective role. Additionally, two long noncoding RNA (lncRNA) located within the Dclk1 locus are overexpressed following MCAO in rats, with a possible role in the epigenetic modifications in the setting of ischemia^70^. However, more research is necessary to better elucidate the function of these proteins following stroke.

*Slc24a4* (also known as NCKX4) encodes a sodium/potassium/calcium exchanger and is immediately 5′ to *Rin3* that is associated with lipid metabolism^71^. *Slc24a4* knockout mice were found to be anorexic, with constitutively activated paraventricular nucleus neurons hypothesized to be secondary to elevated Ca^2+^ signal and melanocortin 4 receptor (MC4R) activity^72^. Melanocyte-stimulating hormone has been shown to have a dose-dependent neuroprotective effect via MC4R signaling following 10 minutes of bilateral carotid occlusion in gerbils via decreased TNF-α and IL-6, as well as decreased MAPK mediated apoptotic pathways^73^. However, this study specifically demonstrated neuroprotection in the hippocampus following global cerebral ischemia and did not investigate ischemic penumbra following stroke. Nevertheless, these findings illustrate a potential mechanism for how SLC24A4 mediates penumbra size. Additionally, SLC24A4 is part of a larger family of ion channels including NCKX2, which has been associated with ischemic brain injury^74^.

SMNTL2 is a downstream target of c-Jun-N-terminal kinases (JNK), predominantly in skeletal muscle although it is expressed in other tissue as well^75^. JNK isoforms facilitate neuron cell death in settings of ischemic stress^76^, and JNK inhibition is neuroprotective following MCAO^77^. However, the function of SMNTL2 is still largely uncharacterized and future study is needed to define its role, if any, in neuroprotection.

Finally, NBEA is a scaffolding protein with a Beige and Chediak-Higashi (BEACH) domain concentrated in neurons that functions in synaptic transmission at the neuromuscular junction^78^ as well as within the central nervous system^79^. Additionally, NBEA loss is associated with both autism and abnormal platelet morphology secondary to large dense core vesicle secretion^80^. Yet, the role of NBEA following stroke is not clear. Similar to SMNTL2, future research targeted at NBEA in stroke is needed.

To further investigate potential interaction amongst these candidate genes, we performed a pathway overrepresentation analysis. Myod1 (MyoD), a helix-loop-helix transcription factor involved in myocyte differentiation^81^, was identified as a regulator of the expression of 4 of the 6 significant genes—*Dclk1, Slc24a4, Nbea*, and *Smtnl2*. Myod1’s canonical role is to induce differentiation of fibroblasts to skeletal muscle cells, but it has also been shown to be expressed in the brain^82^ and may have a role in neural development^83^. Moreover, Dey *et al*. found loss of Myod1 catalyzed sonic hedgehog (Shh) driven neoplastic growth in medulloblastoma^84^. In the setting of ischemia, Shh activity increases in murine neural progenitor cells and neurons^85^. Specifically, the Shh pathway is active in cortex and striatum adjacent to the injured region^86^. Blocking the Shh pathway potentiates brain injury^87^ and Shh agonists have been found to neuroprotective after stroke^88^. The role, if any, of the 4 target genes identified here in Shh signaling is unclear.

Interestingly, the micro RNA miR-145 is associated with multiple proteins identified in the penumbra analysis. DCLK1 post-transcriptionally regulates miR-145 in pancreatic tumor zenografts and DCLK1 knockout mice were found to have increased expression of miR-145^89^. MiR-145 has also been implicated in ischemic stroke. Using an oxygen-glucose deprivation model with primary neuronal cultures, Zheng *et al*. found overexpression of miR-145 lead to decreased expression of *Aqp4*, the gene encoding aquaporin 4 (AQP4), in astrocytes resulting in a protective effect in the setting of ischemia^90^. MiR-145 was also found to be overexpressed in the cortex after transient (1 hour) MCAO and subsequent reperfusion in rats by Dharap *et al*. and antagonism of miR-145 led to increased levels of peri-infarct superoxide dismutase as well as decreased infarct volumes^91^. Moreover, miR-145 expression was found to be elevated within 24 hours of ischemic stroke in human peripheral blood and its expression correlated with the volume of the ischemic infarct as well as with NIH stroke scale score^92^. Furthermore, CLINT1 has also been shown to be inhibited by miR-145, resulting in cell death in a bladder cancer model^93^ and, using TargetScan^94^, SLC24A4 is a predicted target of miR-145 (http://www.targetscan.org/cgi-bin/targetscan/vert_70/targetscan.cgi?mirg = hsa-miR-145). The finding of multiple genes associated with penumbra ratio and miR-145 suggest genetic polymorphisms in these genes may be modifying a common pathway regulating the rate of penumbra infarction (Supplementary Fig. 4). However, further research is necessary to establish and better define the role of miR-145 in the rate of penumbra infarction.

## Limitations

This study has important limitations warranting further discussion. First, we analyzed 6-hour infarct volumes in 215 mice from 33 unique strains, normalized to another 234 mice with 24-hour infarct volumes. Importantly, this method of estimating penumbra volume is a rough estimate as penumbra was not directly measured. These data should be therefore be considered carefully in the context of an estimated penumbra volume. The relatively small sample sizes may limit the statistical power of our study. The ratio of infarct volumes at six over 24 hours utilized approximate penumbra size but is not a direct measure of at-risk tissue and may result in an underestimation of the genetic effects. In addition, it is possible that other variations in blood vessel anatomy not examined in this study could impact the penumbra. It is also possible that serum pH, glucose, and other metabolites not measured in this study could affect penumbra ratio. Moreover, there are varying degrees of occlusion that can confound the results. In addition, the model used in this study, MCAO, may have limited applicability to human patients as it involves a single mechanism and does not capture the different stroke etiologies or the comorbidities commonly found in human patients presenting with ischemic stroke. Although we have minimized the number of animals necessary to achieve sufficient statistical power, a strain survey inherently involves a large number of animals. Nevertheless, these data provide an advantage over human GWAS in that a controlled experimental model, such as the MCAO, is possible whereas it is not in human subjects. Future mechanistic studies are required to investigate the role of the candidate genes identified on penumbral size.

## Conclusions

The size of ischemic penumbra is paramount in determining the extent of salvageable brain following stroke. While prior studies have investigated genetic polymorphisms associated with infarct size, this is the first genome wide association study in mice specifically investigating penumbra size. We report 18 significant SNPs in 6 protein coding genes, including proteins potentially involved in the inflammatory penumbra and neuronal susceptibility to ischemia, which fundamentally make sense but require more research to better define. A better understanding of the genetic underpinnings of penumbral size could inform more personalized application of acute stroke treatment as well as provide the foundation for potential novel therapies.

## Data Availability

The datasets generated during and/or analyzed during the current study are available from the corresponding author on reasonable request.

## Supplementary information

Supplementary Tables and Figures
