# Supplementary material for: A Genome-Wide Analysis of the Penumbral Volume in Inbred Mice following Middle Cerebral Artery Occlusion
Source: Sci Rep. 2019 Mar 25;9:5070. doi: 10.1038/s41598-019-41592-5 (PMC6433893; doi:10.1038/s41598-019-41592-5)
Supplement: Supplementary file 1 — Supplementary Tables and Figures [file 41598_2019_41592_MOESM1_ESM.docx]

**A Genome-Wide Analysis of the Penumbral Volume in Inbred Mice following Middle Cerebral Artery Occlusion**

Robert F. Rudy, BS^1,2^, Nareerat Charoenvimolphan, BA^1^, Baogang Qian, MD^1^, Annerose Berndt, PhD^3^, Robert M. Friedlander, MD^4^, Scott T. Weiss, MD, MS^2,5^, Rose Du, MD, PhD^1,2,5*^

^1^Department of Neurosurgery, Brigham and Women’s Hospital, Boston, Massachusetts, USA

^2^Harvard Medical School, Boston, Massachusetts, USA

^3^Division of Pulmonary, Allergy, and Critical Care Medicine, University of Pittsburgh School of Medicine, Pittsburgh, Pennsylvania, USA

^4^Department of Neurosurgery, University of Pittsburgh School of Medicine, Pittsburgh, Pennsylvania, USA

^5^Channing Division of Network Medicine, Brigham and Women’s Hospital, Boston, Massachusetts, USA

***Corresponding author**

Rose Du, MD, PhD

Department of Neurosurgery

Brigham and Women's Hospital

75 Francis Street

Boston, MA 02115, USA

Email: [rdu@bwh.harvard.edu](mailto:rdu@bwh.harvard.edu)

Phone: 617-732-6600

Fax: 617-734-8342

**Supplementary Materials**

**Supplementary Table 1.**  Number of mice utilized and excluded. Mice that underwent unsuccessful surgery due to arterial puncture resulting in subarachnoid hemorrhage, died during surgery or before 6 hours, or had an aborted surgery due to unsuccessful occlusion were excluded.

| **Strain** | **# with infarct ratio calculation** | **# with SAH or that died during surgery** | **# that died before 6 hours** | **# with surgery aborted due to unsuccessful occlusion*** |
| --- | --- | --- | --- | --- |
| 129S1/SvImJ | 6 | 1 |  | 1 |
| A/J | 6 | 1 |  |  |
| AKR/J | 7 | 1 |  | 1 |
| BALB/cJ | 6 |  | 1 |  |
| BTBR T^+^ tf/J | 6 | 4 |  | 1 |
| BuB/BnJ | 7 | 2 |  |  |
| C3H/HeJ | 6 | 1 | 3 |  |
| C57BL/10J | 6 | 8 |  |  |
| C57BL/6J | 7 | 1 |  |  |
| C57BLKS/J | 6 | 2 |  |  |
| C57BR/cdJ | 6 | 1 |  |  |
| C57L/J | 7 | 7 |  |  |
| CBA/J | 6 | 2 |  |  |
| CE/J | 6 |  |  |  |
| DBA/1J | 6 | 2 |  |  |
| DBA/2J | 6 | 1 | 1 |  |
| FVB/NJ | 6 | 1 |  |  |
| I/LnJ | 8 |  |  |  |
| KK/HlJ | 6 |  |  |  |
| LG/J | 7 | 1 |  |  |
| LP/J | 6 |  | 1 |  |
| MA/MyJ | 6 |  |  |  |
| MRL/MpJ | 6 |  |  |  |
| NOD/ShiLtJ | 6 | 1 |  |  |
| NON/ShiLtJ | 6 | 1 | 1 |  |
| NZO/HlLtJ | 6 |  |  | 2 |
| NZW/LacJ | 14 | 2 |  |  |
| P/J | 6 | 1 | 1 |  |
| PL/J | 7 |  |  |  |
| RIIIS/J | 7 | 3 |  |  |
| SJL/J | 6 |  |  |  |
| SM/J | 6 |  |  |  |
| SWR/J | 6 | 5 |  |  |

*Does not include mice that had SAH or died during surgery

**Supplementary Table 2:** Physiological parameters for each strain of mice included in the study in the 6-hour and 24-hour MCAO groups.

|  | **6-hour MCAO** | | | | **24-hour MCAO** | | | |
| --- | --- | --- | --- | --- | --- | --- | --- | --- |
| **Strain** | **N** | **Mean Age (wks)** | **Mean Weight (gms)** | **Mean MAP** | **N** | **Mean Age (wks)** | **Mean Weight (gms)** | **Mean MAP** |
| 129S1/SvImJ | 6 | 8.5 | 24.4 | 100.8 | 6 | 8.0 | 24 | 104.8 |
| A/J | 6 | 8.3 | 23 | 79.3 | 6 | 8.2 | 22.5 | 79.8 |
| AKR/J | 7 | 9.3 | 28.1 | 84.7 | 7 | 8.7 | 27.5 | 68.7 |
| BALB/cJ | 6 | 8.0 | 24.4 | 76.7 | 7 | 8.0 | 23.6 | 82.7 |
| BTBR T^+^ tf/J | 6 | 8.5 | 33.9 | 61.3 | 6 | 8.3 | 31.6 | 64.2 |
| BuB/BnJ | 7 | 8.0 | 29.6 | 97.6 | 6 | 8.2 | 28.5 | 77.7 |
| C3H/HeJ | 6 | 8.5 | 25.1 | 84.7 | 6 | 8.3 | 23.2 | 87.7 |
| C57BL/10J | 6 | 8.3 | 23.3 | 73.3 | 5 | 8.0 | 18.7 | 86.2 |
| C57BL/6J | 7 | 8.3 | 25.6 | 98.1 | 6 | 8.8 | 23.7 | 96.8 |
| C57BLKS/J | 6 | 8.5 | 21.5 | 95.2 | 7 | 8.4 | 22.7 | 102.1 |
| C57BR/cdJ | 6 | 8.0 | 27.4 | 83.2 | 6 | 8.0 | 26.5 | 79.5 |
| C57L/J | 7 | 8.1 | 24.4 | 78.7 | 6 | 8.3 | 22.9 | 80.7 |
| CBA/J | 6 | 8.2 | 25 | 77.5 | 10 | 8.4 | 25.9 | 77.4 |
| CE/J | 6 | 8.7 | 25.6 | 87 | 7 | 8.1 | 24.4 | 91.4 |
| DBA/1J | 6 | 8.7 | 22.1 | 88.7 | 6 | 8.5 | 21.3 | 86.8 |
| DBA/2J | 6 | 8.2 | 22.9 | 76.3 | 8 | 8.1 | 22.9 | 85.1 |
| FVB/NJ | 6 | 8.3 | 26 | 86.2 | 8 | 8.1 | 24.8 | 82.8 |
| I/LnJ | 8 | 8.0 | 24.7 | 87.6 | 6 | 8.0 | 24.4 | 75.7 |
| KK/HlJ | 6 | 8.0 | 30.5 | 90.3 | 6 | 8.0 | 29.9 | 64.7 |
| LG/J | 7 | 8.0 | 38.6 | 94.4 | 6 | 8.0 | 38.6 | 85.8 |
| LP/J | 6 | 8.3 | 20 | 79.3 | 6 | 8.3 | 20.5 | 90 |
| MA/MyJ | 6 | 8.3 | 21.6 | 90 | 6 | 8.0 | 23.3 | 76.2 |
| MRL/MpJ | 6 | 8.0 | 38.8 | 64.8 | 6 | 8.3 | 38.9 | 81.4 |
| NOD/ShiLtJ | 6 | 8.2 | 25.4 | 77.7 | 6 | 8.5 | 25.6 | 88.2 |
| NON/ShiLtJ | 6 | 8.0 | 29.1 | 84.8 | 7 | 8.0 | 28.1 | 77.9 |
| NZO/HlLtJ | 6 | 8.2 | 42.6 | 95.3 | 8 | 8.4 | 42.8 | 110.4 |
| NZW/LacJ | 14 | 8.1 | 27.7 | 77.5 | 6 | 8.2 | 28.6 | 83.8 |
| P/J | 6 | 8.3 | 20.5 | 76.8 | 6 | 8.2 | 20.1 | 67.7 |
| PL/J | 7 | 8.1 | 21.8 | 76.7 | 7 | 8.3 | 21.4 | 78.4 |
| RIIIS/J | 7 | 8.4 | 20.5 | 94 | 6 | 8.2 | 21 | 87.3 |
| SJL/J | 6 | 8.5 | 24.2 | 83.7 | 9 | 8.1 | 23 | 86.6 |
| SM/J | 6 | 8.3 | 18.3 | 52.2 | 24 | 8.1 | 17.7 | 71.6 |
| SWR/J | 6 | 8.3 | 22.4 | 100.5 | 6 | 8.5 | 23.5 | 96.2 |

MAP = mean arterial pressure, wks = weeks, gms = grams

**Supplementary Table 3:** Variation in covariates at 6 and 24 hours between strains and association with respective normalized infarct volume and penumbra ratio.

|  | **6 Hours** | | | **24 Hours** | |
| --- | --- | --- | --- | --- | --- |
| **Characteristic** | **Interstrain variation (F value, P value)** | **Association with normalized infarct volume (P value)** | **Association with penumbra ratio (P value)** | **Interstrain variation (F value, P value)** | **Association with normalized infarct volume (P value)** |
| Age (weeks) | F = 2.42,  P < 0.001 | 0.86 | 0.45 | F = 1.67,  P = 0.02 | 0.78 |
| Weight (grams) | F = 45.24,  P < 0.001 | 0.11 | 0.45 | F = 48.83,  P < 0.001 | 0.85 |
| Mean Arterial Blood Pressure (mmHg) | F = 2.24,  P < 0.001 | 0.13 | 0.24 | F = 2.47,  P < 0.001 | 0.44 |

Interstrain variation derived from anova of univariate linear regression model.

Association P values derived from univariate linear regression with respective normalized infarct volumes or penumbra ratio.

**Supplementary Table 4:** 24 significant SNPs (FDR < 0.05) associated with 6-hour normalized infarct volume. The SNPs are located within 4 protein coding genes.

| **SNP** | **Chr** | **BP** | **Major/**  **Minor**  **Allele** | **MAF** | **Percent Missing^*^** | **Protein Coding Gene Containing SNP** | **Variant Type^†^** | **Nearby Protein Coding Genes^‡^ (distance BP)** | **P** | **FDR** |
| --- | --- | --- | --- | --- | --- | --- | --- | --- | --- | --- |
| rs31452396 | 3 | 57807198 | C/T | 0.12 | 3.03 |  | Unknown | *Wwtr1* (427366), *Commd2* (351561), Rnf13 (167851), *Pfn2* (155712), *Tsc22d2* (412239) | 4.69x10^-6^ | 2.82x10^-2^ |
| rs3698728 | 4 | 121268127 | T/C | 0.22 | 6.1 |  | Unknown | *Zmpste24* (497281), *Tmco2* (486296), *Rlf* (380438) | 1.05x10^-5^ | 4.58x10^-2^ |
| rs24886510 | 7 | 37543623 | T/C | 0.29 | 0 | *Tshz3* | Intron |  | 4.85x10^-6^ | 2.82x10^-2^ |
| rs6300275 | 7 | 37641208 | T/C | 0.27 | 3.0 |  | Unknown | *Tshz3* (82644), *Zfp536* (462485) | 3.68x10^-6^ | 2.82x10^-2^ |
| rs6333386 | 7 | 37649360 | G/A | 0.29 | 0 |  | Unknown | *Tshz3* (90796), *Zfp536* (454333) | 4.85x10^-6^ | 2.82x10^-2^ |
| rs31482057 | 7 | 37658028 | C/T | 0.29 | 0 |  | Unknown | *Tshz3* (99464), *Zfp536* (445665) | 4.85x10^-6^ | 2.82x10^-2^ |
| rs32358266 | 7 | 37671149 | A/G | 0.29 | 0 |  | Unknown | *Tshz3* (112585), *Zfp536* (432544) | 4.85x10^-6^ | 2.82x10^-2^ |
| rs6392198 | 7 | 37685213 | A/G | 0.29 | 0 |  | Unknown | *Tshz3* (126649), *Zfp536* (418480) | 4.85x10^-6^ | 2.82x10^-2^ |
| rs6394374 | 7 | 37685556 | G/T | 0.29 | 0 |  | Unknown | *Tshz3* (126992), *Zfp536* (418137) | 4.85x10^-6^ | 2.82x10^-2^ |
| rs31948491 | 7 | 37702420 | G/A | 0.29 | 0 |  | Unknown | *Tshz3* (143856), *Zfp536* (401273) | 4.85x10^-6^ | 2.82x10^-2^ |
| rs31606811 | 7 | 37702466 | C/T | 0.25 | 6.1 |  | Unknown | *Tshz3* (143902), *Zfp536* (401227) | 5.29x10^-6^ | 2.91x10^-2^ |
| rs31323802 | 7 | 37716912 | C/T | 0.29 | 0 |  | Unknown | *Tshz3* (158348), *Zfp536* (386781) | 4.85x10^-6^ | 2.82x10^-2^ |
| rs31809019 | 7 | 38131103 | T/A | 0.29 | 0 | *Zfp536* | Unknown |  | 4.85x10^-6^ | 2.82x10^-2^ |
| rs6378965 | 7 | 38143213 | A/T | 0.29 | 0 | *Zfp536* | Unknown |  | 4.85x10^-6^ | 2.82x10^-2^ |
| rs3726954 | 7 | 38150821 | A/G | 0.29 | 0 | *Zfp536* | Unknown |  | 4.85x10^-6^ | 2.82x10^-2^ |
| rs31449206 | 7 | 38158443 | A/G | 0.29 | 0 | *Zfp536* | Unknown |  | 4.85x10^-6^ | 2.82x10^-2^ |
| rs31575983 | 7 | 38176994 | A/G | 0.29 | 0 | *Zfp536* | Unknown |  | 4.85x10^-6^ | 2.82x10^-2^ |
| rs31034636 | 7 | 38185469 | T/C | 0.31 | 6.1 | *Zfp536* | Unknown |  | 7.78x10^-6^ | 3.55x10^-2^ |
| rs32303676 | 7 | 38212840 | G/T | 0.29 | 0 | *Zfp536* | Unknown |  | 4.85x10^-6^ | 2.82x10^-2^ |
| rs31525275 | 7 | 38248405 | A/G | 0.35 | 6.1 | *Zfp536* | Unknown |  | 4.03x10^-6^ | 2.82x10^-2^ |
| rs31182265 | 7 | 38253683 | G/A | 0.31 | 3.0 | *Zfp536* | Unknown |  | 7.01x10^-6^ | 3.34x10^-2^ |
| rs6249168 | 10 | 26329783 | C/A | 0.23 | 3.0 |  | Unknown | *Samd3* (337805), *L3mbtl3* (234792), *Arhgap18* (143444), *Lama2* (371311) | 5.56x10^-6^ | 2.91x10^-2^ |
| rs13472659 | 11 | 45715732 | C/T | 0.18 | 3.0 | *Clint1* | Synonymous Coding | *Lsm11* (26039), *Thg1l* (44613), *Sox30* (78080), *Adam19* (152272), *Nipal4* (245925), *Cyfip2* (291625), *Itk* (422920), *Fam71b* (494329) | 6.23x10^-6^ | 3.11x10^-2^ |
| rs3677406 | 18 | 75599810 | C/T | 0.21 | 6.1 | *Ctif* | Intron | *Lipg* (478893), *Rpl17* (436775), *Dym* (153190), *Smad7* (44221), *Zbtb7c* (380022) | 3.45x10^-6^ | 2.82x10^-2^ |

Chr = chromosome, BP = base pair, FDR = false discovery rate, MAF = minor allele frequency

* Percent of strains missing information at that allele

† SNP function from UCSC mm9 genome browswer

‡ Nearby genes within 500 kbp excluding cDNA and predicted genes

**Supplementary Figure 1:** Power analysis to assess for genome-wide power (p=10^-5^) using 33 inbred of mice with various SNP effects and number of replicates per strain.

**Supplementary Figure 2:** Summary of 6-hour infarct volume by strain and results from genome-wide association analysis. A) Box plot illustrating distribution of normalized infarct volume at 6 hours sorted by strain. B) Manhattan plot for normalized 6-hour infarct volume. C) QQ plot for the normalized infarct volume at 6 hours.





**Supplementary Figure 3:** Association between penumbra ratio and the average number of P1 segments per strain.

**
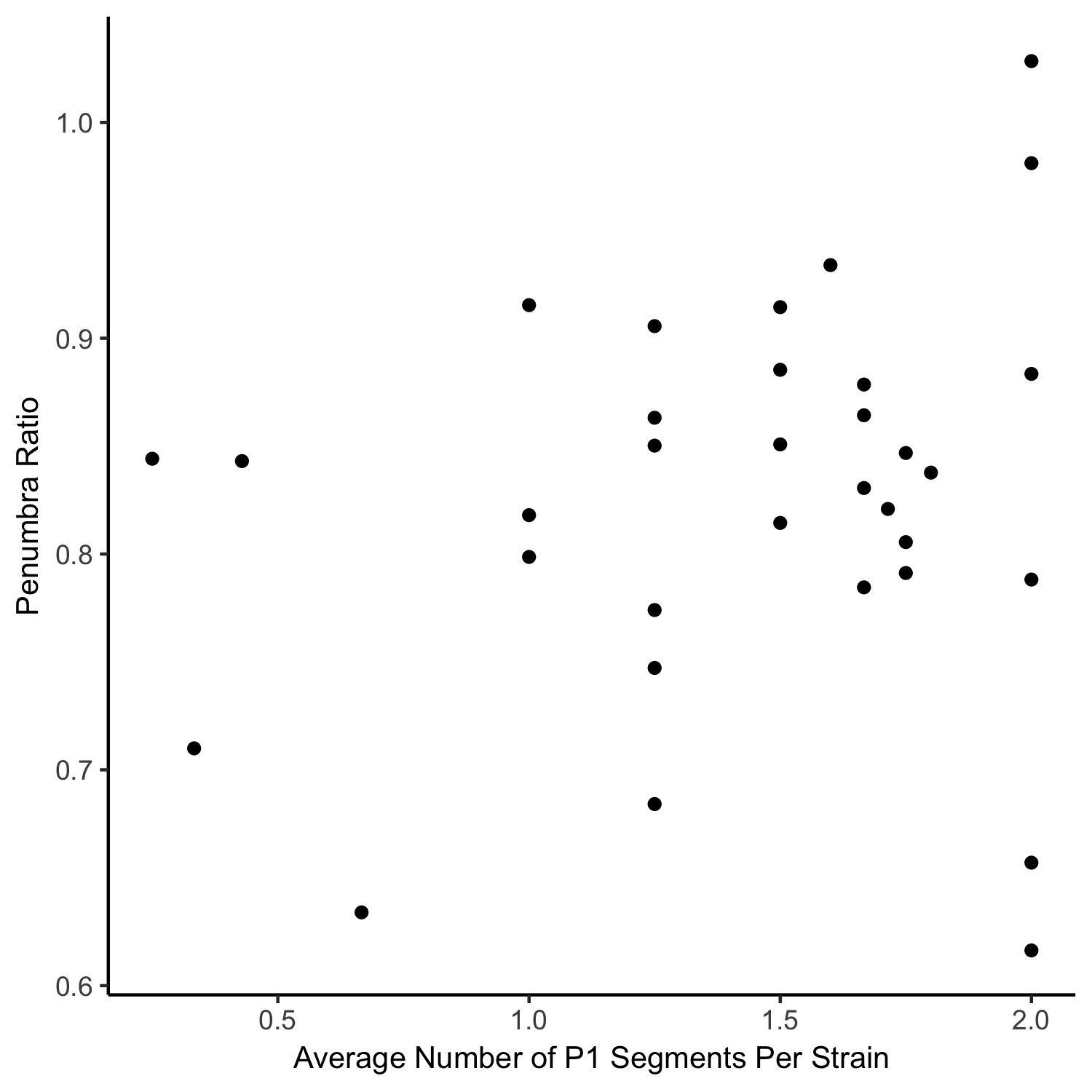
**

**Supplementary Figure 4:** Summary of miR-145 interaction with proteins identified in penumbra analysis (DCLK1, CLINT1, SLC24A4) as well as with two additional proteins found via literature search (AQP4, SOD)

**
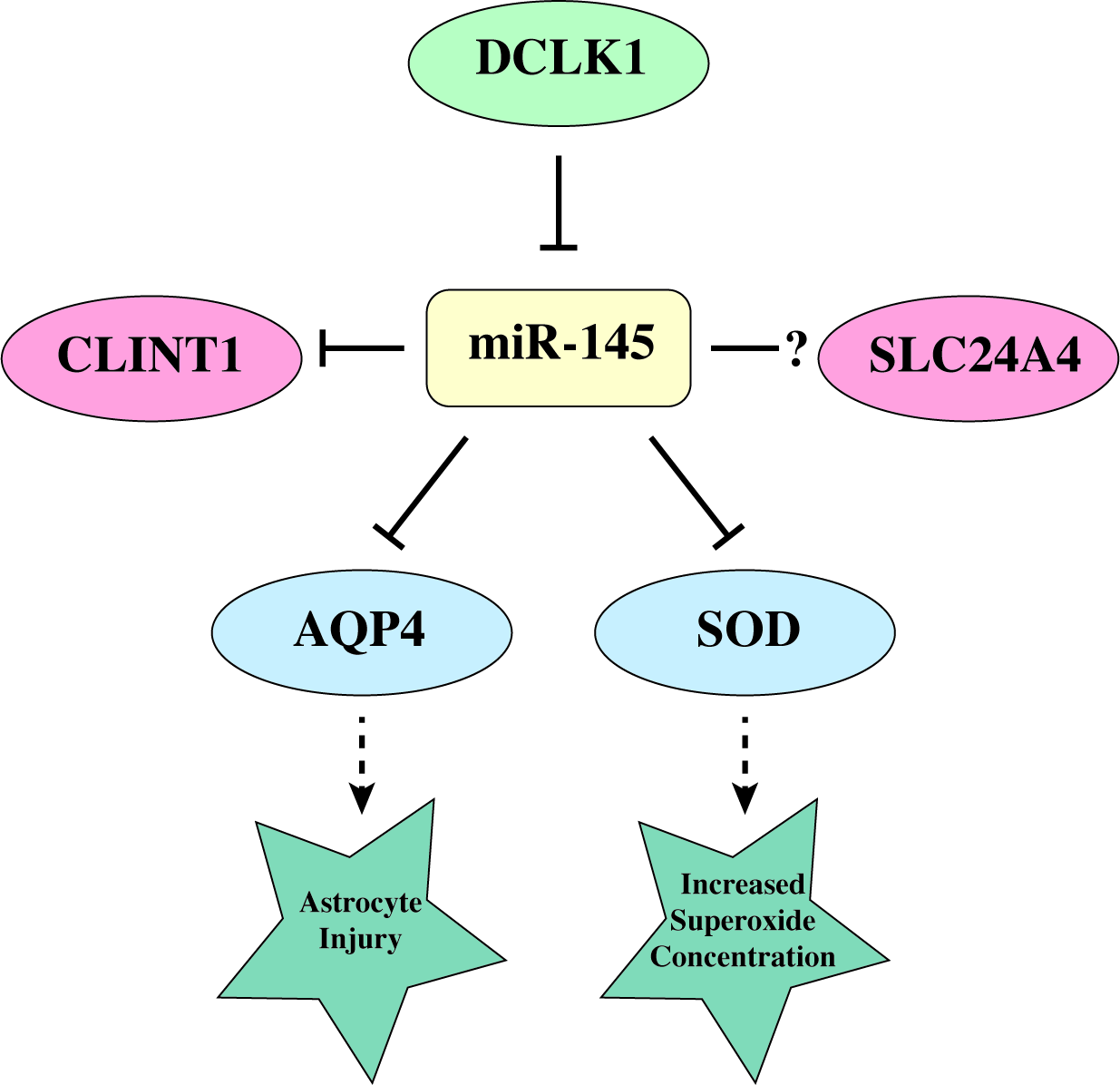
**
